# Growth Promotion and Secondary Metabolites of Vegetables by Spraying Soil with *Psidium guajava, Aloe vera, Allium sativum* and *Medicago sativa* Extracts at Various Stages of Growth

**DOI:** 10.3390/plants14020237

**Published:** 2025-01-16

**Authors:** Ei Ei, Hyun Hwa Park, Yong In Kuk

**Affiliations:** Department of Oriental Medicine Resources, Sunchon National University, Suncheon 57922, Republic of Korea; eieitza@gmail.com (E.E.); camelia9720@nate.com (H.H.P.)

**Keywords:** antioxidative enzyme, growth promotion, plant extracts, secondary metabolites, vegetables

## Abstract

There is a growing need for sustainable, efficient methods to promote plant growth and protect crops, with plant extracts offering natural, multi-component solutions. Based on previous observations, *Psidium guajava*, *Aloe vera*, *Allium sativum* and *Medicago sativa* were selected from 17 water extracts to investigate how the application times of soil sprays affect the antioxidant enzymes and secondary metabolites in fruity and leafy vegetables at different growth stages. From 1 week after sowing (WAS) to 4 WAS, all applications increased the shoot fresh weight by 42–69% in cucumbers, 40–64% in tomatoes, 46–65% in kale and 42–63% in lettuce. These applications also increased the photosynthesis, flavonoids and antioxidative enzymes (ascorbate peroxide (APOD) and guaiacol peroxidase (GPOD)), which provided the plants with a balanced supply of nutrients essential for growth. In the real world, these results show that the use of natural extracts (*P. guajava* and *A. sativum*) can be a sustainable, eco-friendly alternative to synthetic fertilizers and pesticides, helping to improve crop yields and metabolism without harming the environment. This approach could reduce the reliance on chemical inputs and promote more sustainable agricultural practices, especially in controlled environments, like greenhouses, where crops like cucumbers and kale are grown.

## 1. Introduction

Vegetables are a cash crop and are grown to generate income. In order to achieve a large volume and high quality of produce, it is important to use appropriate and improved production practices [1]. Currently, horticulture is facing major challenges when it comes to supplying an ever-growing world population with an adequate amount of nutritious food. The increasing demand for sustainable food, fuel and fiber to curb resource depletion and ecosystem degradation requires the adoption of more sustainable agricultural land management practices. This initiative must focus on reducing input costs and minimizing the reliance on chemical fertilizers and pesticides, as their misuse can pose various risks to human health and the environment. From this perspective, it is important for farmers and researchers to find alternative solutions that increase agricultural productivity while protecting natural resources, especially by minimizing land use [2]. Therefore, alternative and sustainable approaches to solving these problems are being thoroughly investigated. Numerous strategies were proposed, with organic products known as biostimulants being the most intensively researched and promising options to improve the sustainability of agriculture.

The application of plant-derived biostimulants represents an environmentally sound and effective alternative to synthetic counterparts. In addition, plant extracts can be obtained from readily available raw materials, including weeds and other non-commercial plants [3]. They stimulate various physiological processes that improve the uptake and utilization of nutrients by plants. They also promote root development, including the length and number of root hairs, as well as shoot development, yield and nutrient quality. In addition, they counteract the effects of biotic and abiotic stresses; improve the activity of the soil microbiota; and reduce the need for fertilizers and the presence of undesirable compounds, such as nitrates and heavy metals, in crops. The effect of biostimulants can vary depending on plant species; variety; physiological stage; product type; dosage; concentration; timing; and application methods, such as foliar, soil or seed treatments. Additionally, environmental conditions play an important role in these effects [4]. Garlic, for example, was used in organic farming to improve the protection against a variety of diseases [5,6] and to stimulate plant growth [7,8]. In addition, spraying with garlic extract increases the yield and quality of the fruit [9]. Aloe extract (40 mL/L) significantly increased the plant height, number of leaves, number of branches, yield and essential oil content, as well as improved the anatomical structure of the leaves [10]. Guava leaf compost had a better effect on the vegetative growth parameters of Temulawak, such as plant height, stem diameter, and leaf length and width [11]. The aqueous extracts of alfalfa have a significant effect on the root length and yield of beet [1].

Metabolites in the biosystem are roughly divided into primary and secondary metabolites. Primary metabolites are essential for the growth and development of plants, including compounds such as carbohydrates, amino acids, proteins and lipids. In contrast, secondary metabolites (SMs) play a crucial role in improving a plant’s ability to survive and adapt to unfavorable environmental conditions [12]. SMs are often synthesized as bioactive compounds involving specific taxonomic groups of microorganisms [13]. These compounds contribute to plant defense, stress tolerance and ecological interactions.

However, the efficacy of plant extracts containing SMs in biological experiments depends on a clear understanding of how phytochemicals stimulate germination and growth. In particular, their efficacy is usually only observed at low concentrations [14,15]. It is important to note that SMs can have a dual effect. When produced at certain concentrations, they can have phytotoxic effects that may impair the growth and physiological functions of the recipient organisms [16]. Therefore, it is crucial to understand the optimal concentration and application of these metabolites to take advantage of their benefits while avoiding adverse effects.

SMs, such as phenolic acids and flavonoids, which originate from primary metabolic pathways, are essential for plant growth and stress tolerance. These compounds, which are produced during normal growth, exhibit strong antioxidant properties that can be assessed using the 2,2-diphenyl-1-picryhydrazyl (DPPH)-radical-scavenging method [17,18]. There is a strong correlation between the plant antioxidant capacity of a plant and its total phenolic content [19].

Enzymes, such as superoxide dismutase (SOD), catalase (CAT) and peroxidase (POD), play a crucial role in the detoxification of harmful compounds, such as hydrogen peroxide, with POD being involved in lignification, pest defense and wound healing [20]. Plant extracts, such as garlic bulb extract (*Allium sativum*) and *Ascophyllum nodosum*, show significant growth-promoting effects by increasing the fresh and dry weights, photosynthetic pigments, and phenolic and flavonoid contents in treated plants [21,22]. These treatments improve the mobilization of metabolites and shoot development, resulting in faster and more uniform plant growth [23,24].

To better understand the mechanisms by which plant extracts improve crop growth, this study investigated the plant photosynthetic efficiency, as well as DPPH, flavonoids, phenolics and antioxidant enzymes, as these were linked to growth promotion in the above studies. However, much remains to be examined to understand and specify the chemical composition of the extract responsible for the biological activity of selected plant extracts to develop a next-generation environmentally friendly crop protection product for sustainable agricultural production. The research hypothesis confirmed that the effects of analyzing growth and secondary metabolites are still unknown when the plant extracts are applied to vegetable plants at different growth stages.

The current research was therefore an attempt to study the effects of specific water extracts applied at different times as soil sprays on cucumber, tomato, kale and lettuce grown under greenhouse conditions. This study was designed to understand the bio-stimulatory functions of secondary metabolites that contained plant extracts in the growth and defense physiology of vegetable plants.

## 2. Results

### 2.1. Impacts of Selected Water Extracts on Application Timing During Different Growth Phases of Vegetables

**Figure 1:**
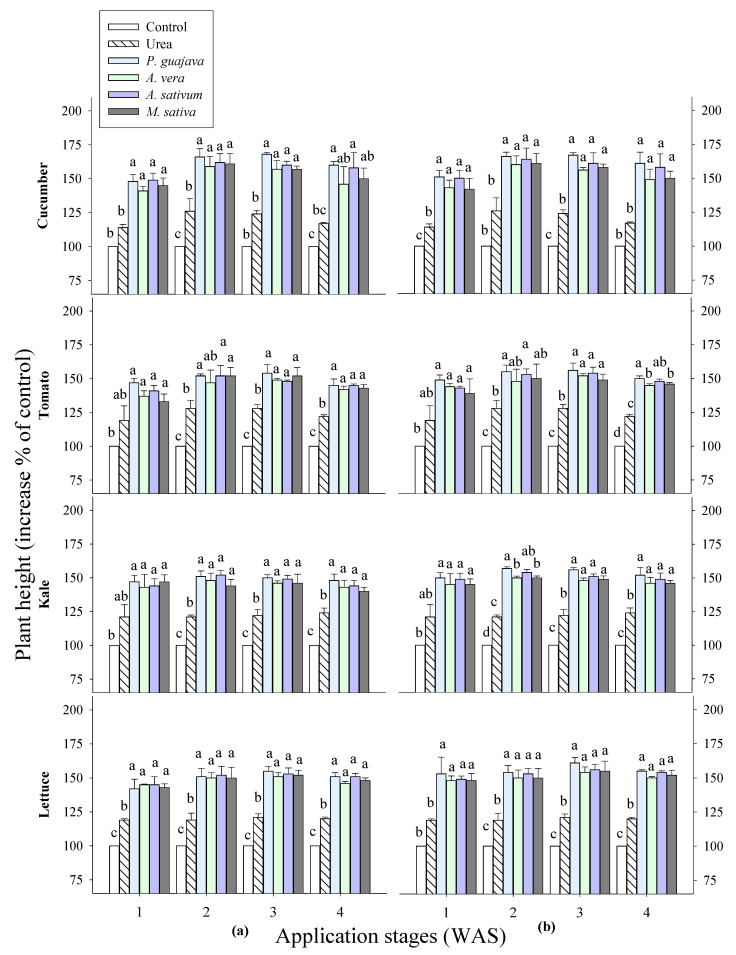
Effects of four water extracts and one urea treatment on the vegetables sprayed at different sowing times: (**a**) 0.1% and (**b**) 0.5% concentrations. Means within bars followed by the same letters did not differ significantly according to Tukey’s HSD at α = 0.05. WAS, week after sowing.

**Figure 2:**
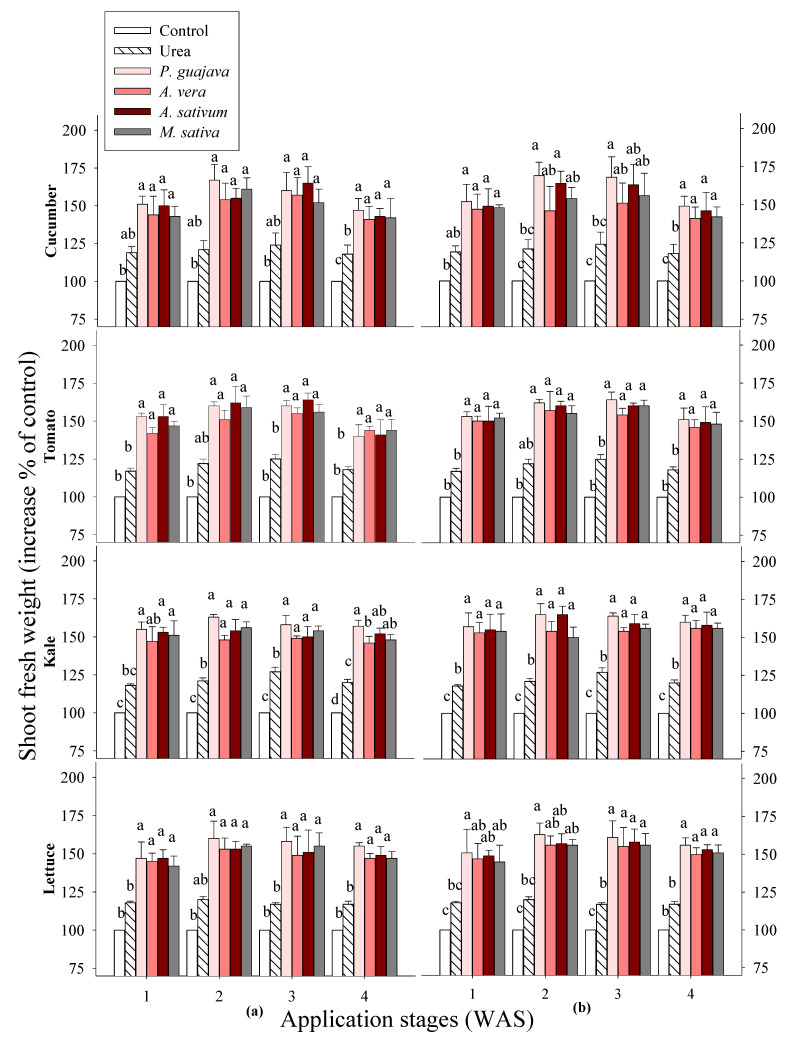
Effects of four water extracts and one urea treatment on the shoot fresh weights of vegetables sprayed at different sowing times: (**a**) 0.1% and (**b**) 0.5% concentrations. Means within bars followed by the same letters did not differ significantly according to Tukey’s HSD at α = 0.05. WAS, week after sowing.

In this study, the growth promotion rates of different vegetables treated with water-based extracts of *Psidium guajava*, *Aloe vera*, *Allium sativum* and *Medicago sativa* at concentrations of 0.1% and 0.5% during different growth stages under greenhouse conditions were investigated. The results of the plant heights and shoot fresh weights of vegetables treated with all the tested extracts indicate significant differences between the crops and application times (Figure 1 and Figure 2). All the test plants showed an increase in the plant height and shoot fresh weight after applying the four water extracts, as the following observations show in comparison with the control group. Consequently, the heights of fruit-bearing vegetables, including cucumber and tomato, increased by 41–50% and 33–49%, respectively, one week after sowing; followed by 58–66% and 46–55% two weeks later; 57–68% and 49–56% three weeks later; and finally, 45–60% and 42–50% four weeks later. The shoot fresh weights of cucumber and tomato increased by 44–52% and 42–53% one week after sowing, by 46–69% and 51–62% two weeks later, by 51–68% and 54–64% three weeks later, and by 42–49% and 40–51% four weeks later. In leafy vegetables, such as kale and lettuce, the application of extracts at one week after sowing led to increases in the plant heights by 43–50% and 42–53%. Two weeks later, the gains were found to be 44–57% and 41–53%. After three weeks, the plant heights showed improvements of 46–56% and 51–60%. Finally, the extracts applied to the plants after four weeks of maturation showed increases of 40–52% and 46–55%. The shoot fresh weights of kale and lettuce increased by 47–57% and 42–51% one week after sowing, by 48–65% and 53–63% two weeks later, by 49–64% and 49–61% three weeks later, and by 46–60% and 47–56% four weeks later. Moreover, the highest shoot fresh weights were observed in all the tested plants in response to the extract of *P. guajava*, followed by the extract of *A. sativum*. At the various growth stages applied, higher growth rates were observed two and three weeks after sowing (WAS). Of the vegetables tested, the cucumber and kale showed the highest growth rates when treated with the selected extracts. Therefore, these growth stages (2 and 3 WAS) were selected to study the secondary metabolites of cucumber and kale treated with extracts of *P. guajava* and *A. sativum*.

### 2.2. Influences of Selected Water Extracts on Secondary Metabolites Production in Vegetables

**Figure 3:**
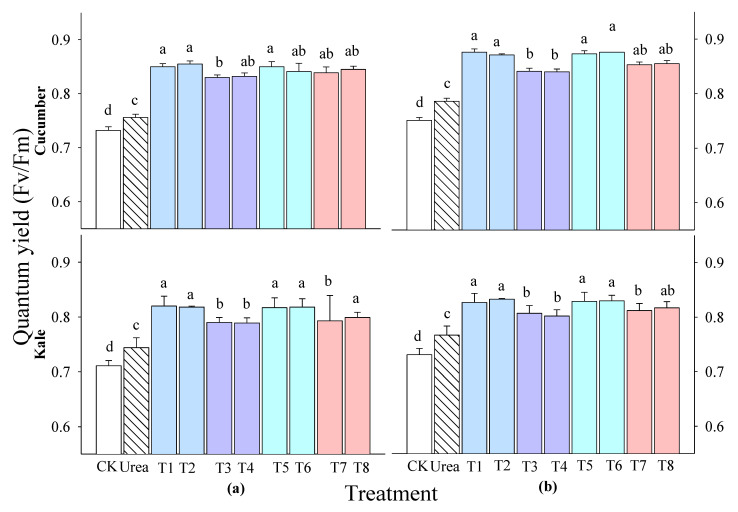
Effects of four water extracts and one urea treatment on the quantum yields of vegetables sprayed at (**a**) 2 weeks after sowing (WAS) and (**b**) 3 WAS. CK, control; T1 and T2, the 0.1 and 0.5% *P. guajava* extracts; T3 and T4, the 0.1 and 0.5% *A. vera* extracts; T5 and T6, the 0.1 and 0.5% *A. sativum* extracts; T7 and T8, the 0.1 and 0.5% *M. sativa* extracts. The parameter was recorded at 7 DAT. Means within bars followed by the same letters did not differ significantly according to Tukey’s HSD at α = 0.05.

Based on the results of the shoot fresh weight, the cucumber and kale plants were selected to evaluate the photosynthetic efficiency at 7 days after treatment (DAT). The photosynthetic efficiency of all vegetables at 2 and 3 weeks after sowing (WAS) varied significantly at 7 DAT (Figure 3). All extract treatments showed better photosynthetic performance than the control and urea treatments. The treatments with extracts of *P. guajava* and *A. sativum* increased the photosynthesis of all tested vegetables more than the treatments with extracts of *A. vera* and *M. sativa*.

**Figure 4:**
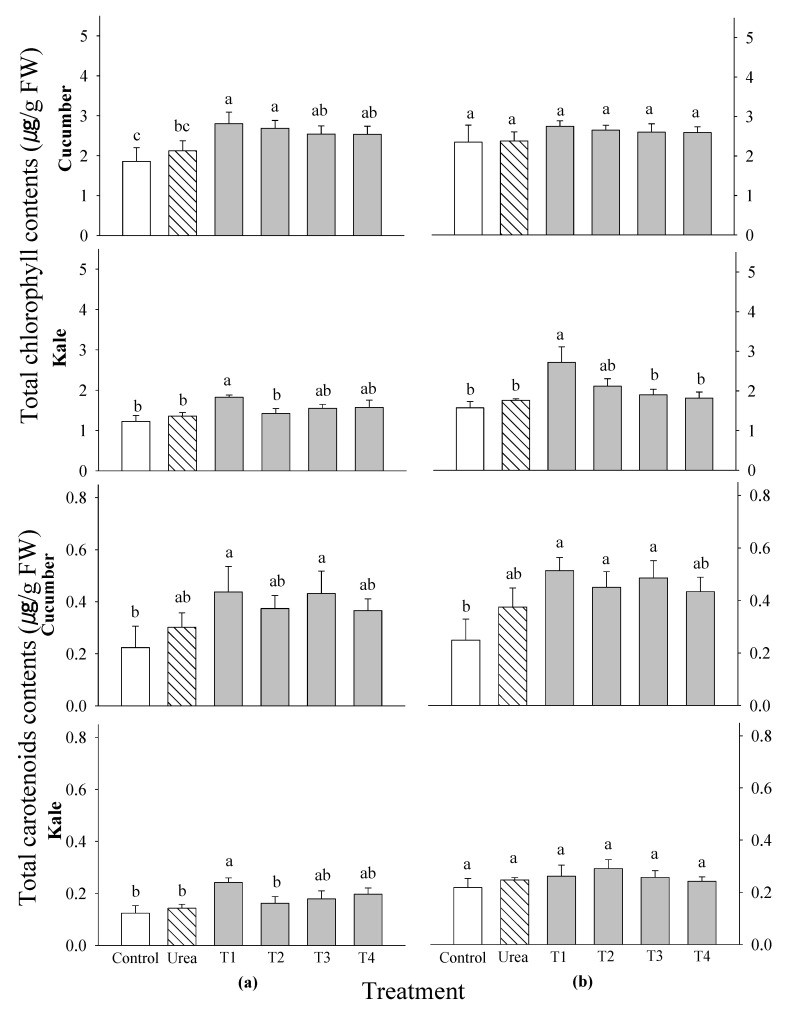
Effects of four water extracts and one urea treatment on the total chlorophyll and carotenoid contents in the vegetables sprayed at (**a**) 2 weeks after sowing (WAS) and (**b**) 3 WAS under greenhouse conditions. T1 and T2, the 0.1 and 0.5% *P. guajava* extracts; T3 and T4, the 0.1 and 0.5% *A. sativum* extracts. Means within bars followed by the same letters did not differ significantly in the LSD test at α = 0.05.

In terms of the total chlorophyll concentrations, cucumber only showed a significant difference at 2 WAS. No significant differences were found in the total chlorophyll contents of cucumber at 3 WAS and kale at both 2 and 3 WAS (Figure 4). Nevertheless, the 0.1% *P. guajava* extract had the highest total chlorophyll compared with the other treatments. The total carotenoid content did not differ significantly between all the treatments across all the tested vegetables and application stages (Figure 4). However, the 0.1% extracts of *P. guajava* and *A. sativum* had the highest total carotenoid content compared with the other treatments. This indicates that they helped to improve the availability of nutrients.

**Figure 5:**
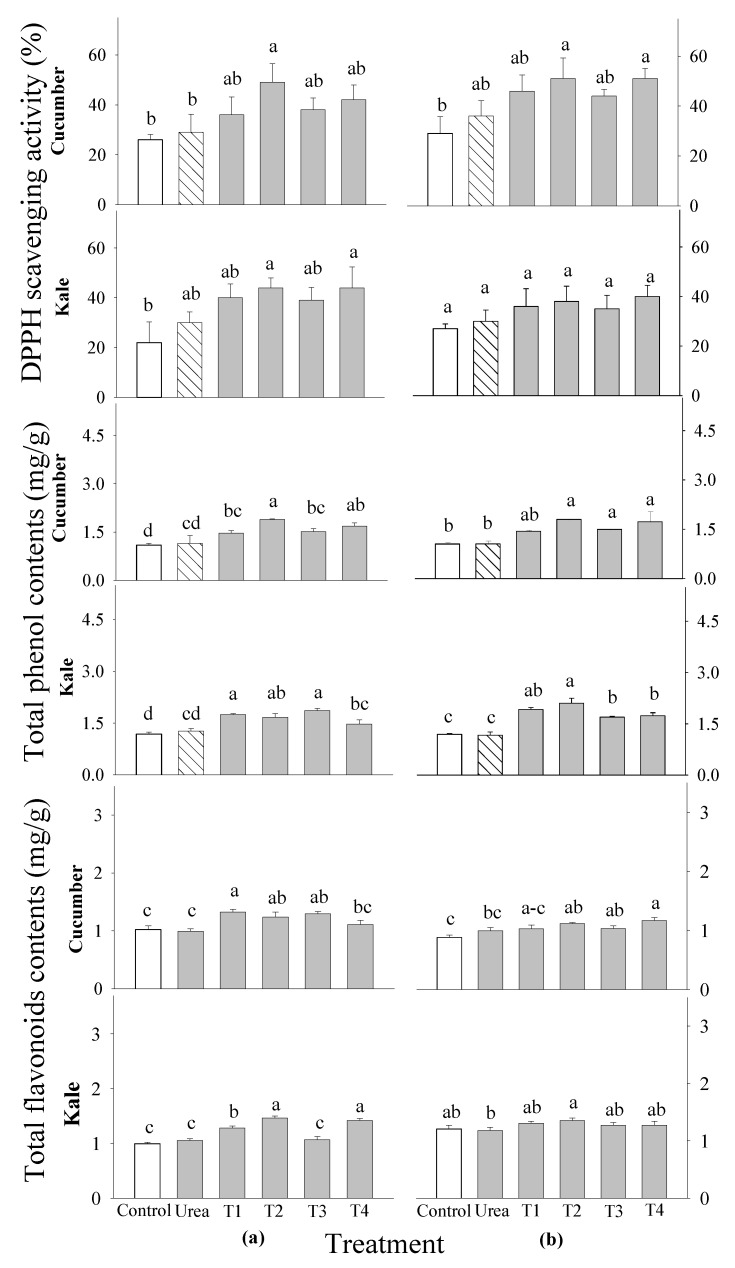
Effects of four water extracts and one urea treatment on the DPPH-radical-scavenging activity and total phenol and total flavonoid contents in the vegetables sprayed at (**a**) 2 weeks after sowing (WAS) and (**b**) 3 WAS under greenhouse conditions. T1 and T2, the 0.1 and 0.5% *P. guajava* extracts; T3 and T4, the 0.1 and 0.5% *A. sativum* extracts. Means within bars followed by the same letters did not differ significantly according to the LSD test at α = 0.05.

The DPPH-radical-scavenging activity of all the tested plants showed significant differences between all the treatment levels and growth stages in the cucumber and kale (Figure 5). The highest concentration was found in the tested vegetables treated with the 0.5% *P. guajava* extract, closely followed by the *A. sativum* extract. The total phenolic and flavonoid contents of the tested vegetables treated with various extract concentrations showed significant differences at 2 and 3 WAS. However, there was no significant difference in the cucumber at 3 WAS (Figure 5). The total phenolic contents were the highest in the cucumber treated with the 0.5% *P. guajava* extract at 2 and 3 WAS. This was followed by the kale treated with the 0.1% *P. guajava* and *A. sativum* extracts at 2 WAS, and finally, the 0.5% *P. guajava* at 3 WAS. The cucumber plants showed the highest flavonoid contents in the 0.1% *P. guajava* treatment at 2 WAS and in the 0.5% *A. sativum* treatment at 3 WAS. In contrast, the kale plants showed their highest flavonoid contents with the 0.5% *P. guajava* treatment at both 2 and 3 WAS.

**Figure 6:**
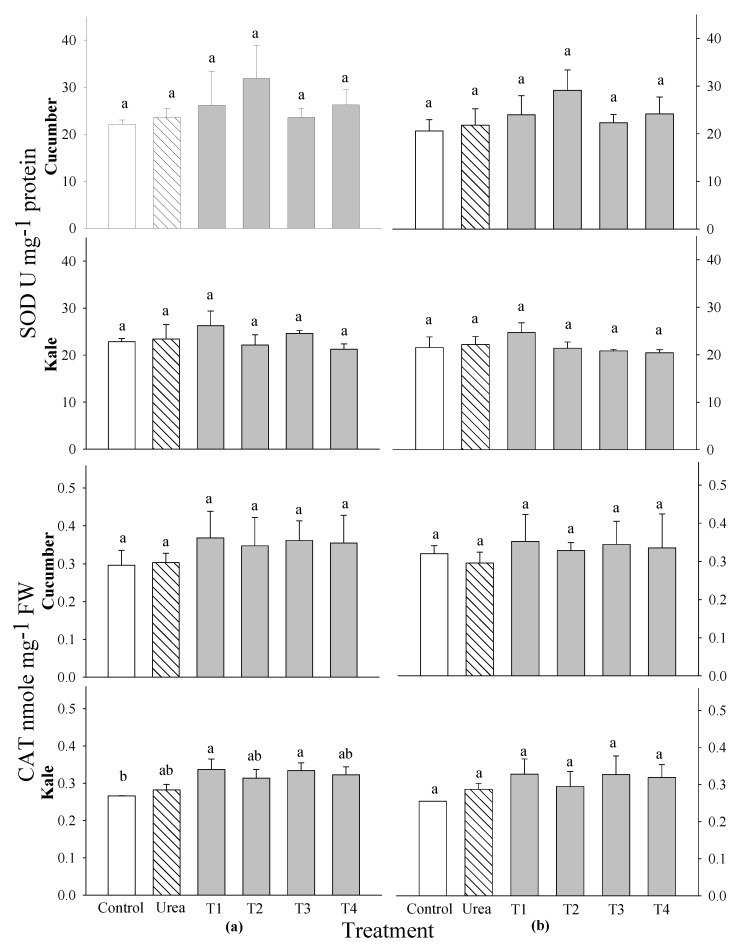
Effects of four water extracts and one urea treatment on the SOD and CAT activities in vegetables sprayed at (**a**) 2 weeks after sowing (WAS) and (**b**) 3 WAS under greenhouse conditions. T1 and T2, the 0.1 and 0.5% of *P. guajava* extracts; T3 and T4, the 0.1 and 0.5% *A. sativum* extracts. Means within bars followed by the same letters did not differ significantly according to the LSD test at α = 0.05.

**Figure 7:**
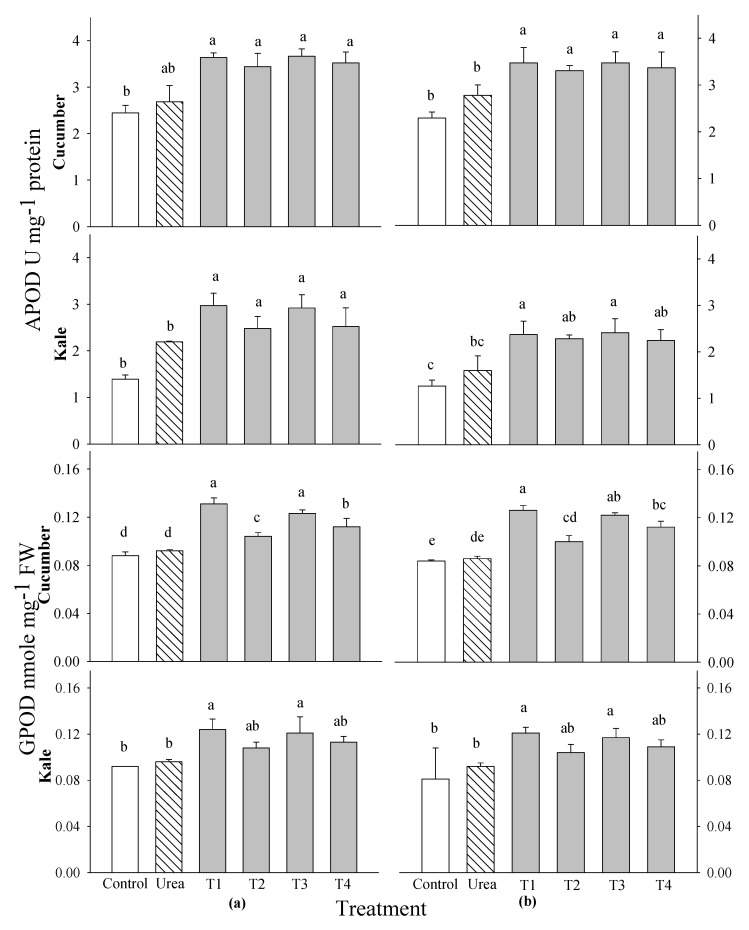
Effects of four water extracts and one urea treatment on the APOD and GPOD activities in the vegetables sprayed at (**a**) 2 weeks after sowing (WAS) and (**b**) 3 WAS under greenhouse conditions. T1 and T2, the 0.1 and 0.5% *P. guajava* extracts; T3 and T4, the 0.1 and 0.5% *A. sativum* extracts. Means within bars followed by the same letters did not differ significantly according to the LSD test at α = 0.05.

At 2 and 3 WAS, the activities of superoxide dismutase (SOD), catalase (CAT), guaiacol peroxide (GPOD) and ascorbate peroxide (APOD) were tested in cucumber and kale plants after the addition of the 0.1% and 0.5% extracts of *P. guajava* and *A. sativum*. The activities of SOD and CAT in the cucumber and kale plants did not differ significantly from the control group at any of the application stages (Figure 6). The highest SOD activities were observed in the cucumber treated with the 0.5% *P. guajava* extract and in the kale treated with the 0.1% *P. guajava* extract at 2 and 3 WAS. The highest CAT activities were observed in the cucumber and kale treated with the 0.1% *P. guajava* and *A. sativum* extracts. However, there were notable significant differences in the APOD activities compared with the control in all vegetables and different stages of application (Figure 7). The most significant APOD activities were observed in the cucumber and kale treated with the 0.1% *P. guajava* and *A. sativum* extracts at 2 and 3 WAS. Nonetheless, the GPOD activities of the cucumber showed a remarkable difference compared with the control at 2 and 3 WAS (Figure 7). However, no significant difference in the GPOD activities were observed in the kale at both 2 and 3 WAS. The highest GPOD activities were observed with the 0.1% extracts of *P. guajava* applied to the cucumber and kale plants at each application stage.

Among the different treatments, all extract applications resulted in a greater improvement of secondary metabolites in the cucumber and kale compared with the urea treatment. The degradation of DPPH radicals, phenols, flavonoids and antioxidant enzymes (SOD, CAT, APOD and GPOD) was higher in the plants that received the 0.1% water extracts of *P. guajava* and *A. sativum* than in the control and other treatments.

## 3. Discussion

The practice of spraying soils with water extracts is widespread. However, the effects of the timing of these applications have not yet been systematically investigated. In this study, we found that the stages of soil spraying with water extracts had both synergistic and antagonistic effects on the growth of fruity and leafy vegetables and on the physiological properties related to secondary metabolites, including photosynthesis, chlorophyll, carotenoid, phenolic, flavonoid, DPPH-radical-scavenging and antioxidant enzyme activities.

The results in Figure 1 show that the growth of fruity and leafy vegetables was significantly improved by the application of the water extracts of *Psidium guajava*, *Aloe vera*, *Allium sativum* and *Medicago sativa*, as evidenced by the plant height and shoot fresh weight. The results of other studies agree with this study that the compost of guava leaves (*P. guajava*) gives the best development of the Temulawak plant (*Curcuma xanthorrhiza* Roxb). This is supported by the fact that guava leaves are rich in nitrogen, phosphorus and potassium, but the concentrations of these elements vary depending on the month in which the plant is growing [25]. Based on the *A. sativum* extracts, other studies agree with our study that the highest values for plant height, stem diameter, dry weight of leaves per plant, leaf area, total carbohydrates and nitrogen content were obtained in *Schefflera arboricola* with garlic extract [26]. Moreover, treatment with garlic extract improved the growth parameters of pepper plants [27]. A study on cucumber plants showed that the foliar application of garlic extracts at a concentration of 2.5 mL/L resulted in a remarkable increase in plant length, leaf number, leaf area, total chlorophyll content in leaves, nodule percentage and total soluble solids. This improvement has been linked to the water extract of the garlic plant, which consists of 30% carbohydrates and is rich in potassium, iron, magnesium, phosphorus, thiamine, riboflavin, niacin, ascorbic acid and essential oils [28]. All of these compounds play a role in photosynthesis by promoting its products and the Krebs cycle. Together, they contribute to the formation of the most important compounds in the plant and improve its physiological properties.

The results also show an increase in the photosynthetic pigments of the cucumber and kale at 2 and 3 WAS (Figure 2). The results clearly confirm that the extracts of *P. guajava* and *A. sativum* increased the photosynthetic rate of rice at 7 days after treatment [29]. The increase in the metabolic activity of quinoa with garlic extracts may have been associated with a corresponding increase in the photosynthetic pigments, which subsequently increased the carbohydrate content, in addition to the increase in the protein content due to the higher nitrogen content [30]. Another finding [11] states that the use of fertilizers, such as water extracts, ensures that plants have enough building blocks to carry out photosynthesis. The amount of light and chlorophyll significantly influences the photosynthetic process, reinforcing the idea that plants with longer and wider leaves produce a more vigorous rhizome. In addition, a stimulated photosynthetic system indicates an enhanced ability to produce food [31,32], potentially impacting the plant growth. However, a significant difference in the total chlorophyll content of the cucumber was found at 2 WAS (Figure 3). Garlic root exudates increase the chlorophyll content and thereby increase the absorption of light energy in tomatoes and peppers, leading to an increased photosynthesis rate [33]. This was in line with a study that showed that the application of 4% garlic extract to pear plants increased the nitrogen and potassium contents in the leaves, which is known to increase the chlorophyll concentration [9]. The results clearly confirm previous studies indicating that the chlorophyll content was influenced by various treatments and sometimes improved development and physiological conditions [34,35,36,37]. In this study, the data show strong correlations between the vegetable production parameters, such as plant height, shoot fresh weight and photosynthetic rate, emphasizing the importance of these three parameters as valuable indicators of plant productivity.

An optimal extract should have no detrimental effect on the quality of the crop and, at the same time, be an effective growth promoter. Therefore, the contents of phenols and flavonoids, as well as the activities of the DPPH radical scavenging and the antioxidant enzymes, in the leaves of the cucumber and kale plants were investigated. The plants that received the 0.1% water extracts of *P. guajava* and *A. sativum* showed increased levels of DPPH radical scavenging, phenolic acids, flavonoids and antioxidant enzymes (SOD, CAT, APOD and GPOD) compared with the control and the other treatments (Figure 5, Figure 6 and Figure 7). Results similar to ours indicate that there were significant differences in DPPH radicals scavenging and flavonoid content in rice treated with guava and garlic extracts [29]. In addition, the total phenol and flavonoid contents and DPPH-radical-scavenging activity in the leaves of *Peucedanum japonicum* showed increases of 23–81% compared with the control when treated with various extracts, including Chinese chive and soybean leaf extracts, at concentrations ranging from 0.5 to 3% [38]. The antioxidant activity of extracts evaluated using DPPH assays exhibited a good correlation with the total phenolic and flavonoid content [39]. Moreover, it was discovered that certain phenolic acids at low concentrations increased the dry weight, number and length of secondary roots, and the length of main roots in *Deschampsia flexuosa* and *Senecio sylvaticus* [40]. According to another study, cucumbers treated with fermented bone + fish extracts had a much higher total flavonoid content than the control plants, whereas plants treated with other extracts produced fruit with flavonoid contents comparable with the control plants [41]. The antioxidant properties of the selected plant extracts may be mostly attributable to the phenolic and flavonoid groups [42].

Spraying with the extracts of *P. guajava* and *A. sativum* improved the activities of antioxidant functions in this study. The actions of APOD and GPOD were examined at 2 and 3 WAS in the cucumber and kale after utilizing *P. guajava* and *A. sativum* at 0.1% concentrations (Figure 6 and Figure 7). These results are consistent with the studies of [43], which indicate that AGE had biological activity in cucumber seedlings and altered plant defense mechanisms, probably through the activation of relative oxygen species at low concentrations. Similar to our findings, other studies also indicate that APOD and GPOD levels increased in rice treated with guava and garlic extracts [29]. Further studies showed that the expressions of SOD and POD were stimulated by stress, which probably led to increased ROS levels in eggplant seedlings. A balance of ROS levels could prevent lipid peroxidation, maintain cellular homeostasis [44] and ultimately promote eggplant growth. Garlic serves as an antioxidant that can modulate these processes [45].

In our experiment, the extracts with increased total phenolic and flavonoid contents, including P. guajava and A. sativum, showed improved DPPH-scavenging capacity, highlighting the significant contribution of polyphenols to antioxidant enzyme activities (APOD, GPOD), which provides additional evidence for the role of polyphenolic compounds in strengthening plant defense mechanisms. The observed increase in these enzymes after treatment with polyphenol-rich extracts suggests that the polyphenolic compounds play a dual role by both directly scavenging free radicals and enhancing the plant’s inherent antioxidant defense mechanisms. Comparable observations were documented by [46], who indicated that polyphenol-rich extracts contribute to the enhancement of antioxidant enzyme activities in *A. sativum*. These results agree with the findings of [47], who indicated that the antioxidant potential of *P. guajava* is closely related to its polyphenol content, which plays an important role in both the DPPH-scavenging activity and the enhancement of enzymes. Consequently, the observed positive relationship between the total phenolic and flavonoid contents and the antioxidant activity highlights the important role that polyphenolic compounds play in the antioxidant defense mechanisms of plants.

Fortunately, the growth-promoting extracts used in this study had no negative effects on the secondary metabolites. All the extracts showed growth-promoting effects at optimal doses, with *P. guajava* and *A. sativum* being the most effective [29]. It is known that guava leaf contains flavonoids, sesquiterpenes, triterpenoids, coumarins, alkaloids and tannins. In addition, research demonstrated numerous physiological activities of concentrated *P. guajava* leaf extract, including the ability to inhibit hyperglycemia, exhibit antioxidant properties, exhibit antibacterial effects and inhibit tyrosinase activity [20]. Organosulfur compounds derived from garlic, in particular allicin, diallyl disulfides and diallyl trisulfide, are known for their potent antioxidant properties and their ability to actively interact with lipid bilayers [48]. Consequently, they may play a role in signaling and altering the physiochemical properties of recipient plants. Therefore, these extracts demonstrate potential as safe and natural herbal stimulants for enhancing the plant growth.

The plant extracts (*P. guajava, A. vera, A. sativum, M sativa*) showed significant improvements in plant growth by increasing the plant heights by 33–68% and shoot fresh weights by 40–69% compared with the control group. In contrast, the treatment with urea only resulted in 20% increases in both values, meaning that the plant extracts resulted in 13–48% increases in the plant heights and 20–49% increases in the shoot fresh weights compared with urea. In addition, the extracts, particularly *P. guajava* and *A. sativum*, significantly increased the photosynthesis, total chlorophyll, carotenoid content, DPPH-scavenging activity, total phenolic and flavonoid contents, and antioxidant activity compared with the urea treatment, indicating their superior impact on both growth and biochemical composition.

Compared with other commercial biostimulants, including humic-acid-based products and amino acid formulations commonly used to improve growth and stress resistance, the plant extracts showed superior efficacy. Humic acid compounds generally promote growth by 10–30% and improve nutrient uptake and root formation [49]. Amino acid formulations often provide a 15–25% increase in growth, mainly by improving nitrogen assimilation and protein synthesis [50]. In contrast to urea, which is widely used in agriculture and is known to improve plant development by 15–25% [51], the plant extracts used in this work showed an improvement of over 50% in growth metrics and biochemical properties. The influence of urea on plants is primarily limited to the nitrogen supply, while plant extracts have a more holistic growth-promoting effect due to their diverse bioactive components. Compared with conventional biostimulants, including algae extracts and humic-acid-based solutions, which are known to improve growth and antioxidant properties by 20–40% [52], the plant extracts used in this study showed better results. Biostimulants from algae and humic acid generally improve the chlorophyll content and overall plant vigor via cytokinin- and auxin-like mechanisms [53]. Nevertheless, the plant extracts showed superior performances due to their rich bioactive compounds, making them sustainable and highly effective alternatives.

## 4. Materials and Methods

### 4.1. Preparing Plant Materials and Extracts

A variety of vegetables were utilized as test crops, including cucumbers (*Cucumis sativus*) (cv. Ho Dong Cheong Jang F1), tomatoes (*Solanum lycopersicum*) (cv. Dotaerang Myeongpum), kale (*Brassica oleracea* var. *sabellica*) (cv. Saeron kale) and lettuce (*Lactuca sativa*) (cv. Cheong Ha Cheong Chi Ma). These vegetable seeds were purchased from Asia Seed Korea Co., Ltd. (Seoul, Korea). Plants of the species guava (*Psidium guajava*), aloe (*Aloe vera*)*,* garlic (*Allium sativum*) and alfalfa (*Medicago sativa*) were used to produce the water extracts that were utilized in this investigation. In a previous study [29], these water extracts showed the highest rates of enhancement in rice seedling development among 17 tested extracts, which led to their selection.

### 4.2. Conditions for the Cultivation of Plants and Their Treatment

To cultivate the vegetables, approximately 200 g of commercial soil for horticultural media (No. 2 Sunghwa, Republic of Korea) was placed inside each pot (16 cm in height and 16 cm in diameter). The composition of these commercial soils consisted of zeolite 5%, perlite 8%, peat moss 8%, vermiculite 12%, cocopeat 66.6% and fertilizer 0.4%. The seeds were sown in a 50-cell plastic tray with commercial soil. After the germination of the seedlings, one seedling of each vegetable was put in each pot at the two-to-three-leaf stage. The growth-enhancing effects of four water extracts were examined during various growth stages of all the crops [29]. The application times were different for each growth stage (1, 2, 3- and 4-weeks post-sowing). Three to five days after transplanting, four different extracts (10 mL per pot) were applied into the soil at amounts of 0.1 and 0.5% according to the results of a previous study [29]. Distilled water served as the control treatment. Urea at 0.6% was used as a benchmark for assessing the growth efficacy of the extracts. The pots were maintained in a greenhouse for two weeks following the extract treatments. The greenhouse conditions were 14 h of illumination and 10 h of obscurity, with diurnal/nocturnal temperatures of 30 ± 2 °C/20 ± 3 °C, 70% relative humidity and a photosynthetically active radiation (PAR) of 500 µmol m^−2^ s^−1^. A measurement of the shoot fresh weight was taken at 14 days after treatment (DAT). All studies were conducted in duplicate, with three duplicates for each treatment.

### 4.3. Impacts of Specific Water Extracts on Secondary Metabolite Synthesis in Vegetables

Samples were taken from three plants in each of the three groups at two and three weeks following the extracts’ application. The leaf samples were promptly frozen in liquid nitrogen and preserved at −80 °C for later biochemical investigation. In this study, the secondary metabolites were analyzed in terms of the photosynthesis efficiency, total chlorophyll and carotenoid contents, DPPH-scavenging activity, total phenolic and flavonoid contents, and antioxidant enzyme contents (SOD, CAT, APOD and GPOD).

#### 4.3.1. Determination of Quantum Yield, Total Chlorophyll and Carotenoid Contents

The chlorophyll α fluorescence of photosystem II (PSII), specifically the quantum yield (Fv/Fm) of the rice, cucumber and kale crops, was assessed at 7 days after treatment (DAT). A soil drench was used to administer 10 mL of water extracts at concentrations of 0.1 and 0.5% to each plant for a period of 2 and 3 weeks after sowing (WAS). The second leaves of the rice, cucumber and kale plants were chosen at 7 DAT to be measured with a portable pulse modulation fluorometer (Fluorpen FP10, Photon Systems Instruments, Drásov, Czech Republic). Prior to the measurements, the fronts were darkened for 15 min to ensure the activation of all antenna pigments.

Analyses of chlorophyll and carotenoids were conducted following a previously established method [54]. Seedling leaves (0.5 g) from each treatment group were homogenized in a 100% methanol solution. The extracts underwent centrifugation at 10,000× *g* for 3 min, after which the absorbance of the supernatant was spectrophotometrically measured at 470, 652 and 665 nm. The calculation of chlorophyll and carotenoid contents was performed using the following equations:

Chlorophyll a (C_a_) = 16.72 A_665.2_ − 9.16 A_652.4_

Chlorophyll b (C_b_) = 34.09 A_652.4_ − 15.28 A_665.2_

Total chlorophylls (C_a+b_) = 1.44 A_665.2_ + 24.93 A_652.4_

Total carotenoids (C_x+c_) = [1000 A_470_ − 1.63 C_a_ − 104.96 C_b_]/221

#### 4.3.2. Determination of DPPH-Radical-Scavenging Activity and Total Phenol and Flavonoid Contents

The total phenol and flavonoid levels, as well as the DPPH-radical-scavenging activity, were detected using a method that has been reported in [55]. The dried plant samples (0.5 g) were combined with 10 mL of 99.9% ethanol, shaken at 120 revolutions per minute (rpm) for 24 h at a temperature of 27 °C in a shaking bath and centrifuged at 13,000 rpm for 10 min using a high-speed refrigerated centrifuge (VS-24SMTI, Vision Scientific Co., Ltd., Daejeon, Republic of Korea); these activities were then analyzed.

To assess the DPPH radical scavenging activity, a mixture was prepared consisting of 0.1 mL of the extract, 0.5 mL of a 0.1 M acetate buffer solution (pH 5.5), 0.25 mL of 0.5 mM DPPH (2,2-diphenyl-1-picrylhydrazyl) and 0.4 mL of ethanol. This mixture was incubated at room temperature for 30 min. The solution was examined with a 517 nm of UV spectrometer (SPECTROstar Nana, BMG LABTECH, GmbH, Offenburg, Germany) to determine the constituents. Ascorbic acid served as a positive control, and the DPPH-radical-scavenging activity of the extracts was determined using the following formula:

DPPH radical scavenging activity (%) = [Ac − As/Ac] × 100

Ac represents the absorbance of the control, while As denotes the absorbance of the test samples [56].

After combining 0.2 mL of the extract with 0.6 mL of distilled water and 0.2 mL of Folin–Denis reagent, the mixture was agitated for 5 min to ascertain the total phenol concentration. The solution was combined with 0.2 mL of Na_2_CO_3_ and left at room temperature for 1 h after 5 min. Then, it was measured at 640 nm using a UV spectrophotometer (SPECTROstar Nana, BMG LABTECH, GmbH, Offenburg, Germany). The standard values were derived from ferulic acid concentrations that ranged from 0 to 100 µg/mL, and the total phenol values were determined using a standard curve. Each value is presented as a mean (mg of ferulic acid equivalents per g of extracted sample).

The total flavonoid content was measured by combining 0.2 mL of the extract with 0.45 mL of 95% ethanol, 0.0.3 mL of 10% AlCl_3_, 0.03 mL of 1 M potassium acetate and 0.79 mL of distilled water. The mixed solution was allowed to stand for 40 min at room temperature, and the absorbance was measured at 640 nm using a UV spectrophotometer (SPECTROstar Nana, BMG LABTECH, GmbH, Offenburg, Germany). The standard values were derived using quercetin acid at 0–5 µg/mL concentrations, and the total flavonoid values were determined based on a standard curve. Each value is presented as a mean (mg of quercetinic acid equivalents per g of extracted sample).

#### 4.3.3. Determination of Antioxidative Enzyme Activities

To obtain the enzymes from the frozen leaves, about 0.5 g of them were mixed with 3.75 mL of a 100 mM buffer solution (pH 7.5) that had 2 mM EDTA, 1% PVP and 1 mM phenylmethylsulfonyl fluoride (PMSF, C_7_H_7_FO_2_S). The mixture was then broken up with a mortar and pestle and put in a refrigerated centrifuge (VS-24SMTI, high-speed refrigerated centrifuge, Vision Scientific Co., Ltd., Daejeon, Republic of Korea) at 14,000× *g* for 20 min.

Superoxide dismutase (SOD) activity was assessed using the method outlined in [57], which incorporated several modifications [58,59]. A reaction mixture of 230 µL comprised 1.26 mM NBT (Nitroblue tetrazolium), 260 mM riboflavin, 260 µM methionine, 2 mM EDTA (Ethylenediamine tetraacetic acid) and 200 mM phosphate buffer at pH 7.0, supplemented with 20 µL of enzyme extract, which resulted in a total volume of 300 µL. The mixture in the test tubes was exposed to light at an intensity of 78 mmol photon s^−1^ m^−2^ for 10 min, after which the absorbance at 560 nm was measured. A nonirradiated reaction mixture that did not exhibit color functioned as the control, and its absorbance was subtracted from A560 of the reaction solution. One unit of SOD activity is defined as the quantity of enzyme necessary to achieve 50% inhibition of the NBT reduction rate at 560 nm (SPECTROstar Nana, BMG LABTECH, GmbH, Offenburg, Germany).

The activities of catalase (CAT) and guaiacol peroxidase (GPOD) were determined by employing a method that has been published in [60,61]. Spectrometer readings taken at 240 nm for 1 min (SPECTROstar Nana, BMG LABTECH, GmbH, Offenburg, Germany) were used to determine the CAT activity by measuring the rate of H_2_O_2_ breakdown. The reaction mixture comprised 150 µL of 200 mM phosphate buffer (pH 7.0), 15 µL of 15 mM H_2_O_2_, 120 µL of distilled water and 15 µL of enzyme extract, which resulted in a total volume of 300 µL that initiated the reaction. The oxidation of guaiacol during GPOD activity was assessed by monitoring the increase in the absorbance at 470 nm over a duration of 1 min (SPECTROstar Nana, BMG LABTECH, GmbH, Offenburg, Germany). Ingredients in the reaction mixture included 5 µL of 200 mM guaiacol, 280 µL of 200 mM phosphate buffer (pH 7.0) and 15 µL of enzyme extract. The reaction commenced with the addition of 5 µL of 40 mM H_2_O_2_.

The ascorbate peroxide (APOD) activity was determined by its absorbance at 290 nm at the beginning rate of reduction in the ascorbate concentration [62]. The total volume of the reaction mixture was 300 µL, and it consisted of 150 µL of 200 mM phosphate buffer (pH 7.0), 30 µL of 5 mM ascorbate, 12 µL of 20 mM H_2_O_2,_ 30 µL of distilled water and 15 µL of enzyme extract. The addition of H_2_O_2_ was the catalyst that started the reaction, and a UV spectrophotometer (SPECTROstar Nana, BMG LABTECH, GmbH, Offenburg, Germany) was used to quantify the change in the absorbance.

### 4.4. Experimental Design and Statistical Analysis

The experiment was set up with a factorial layout and three replicates in a completely randomized design. Multiple comparisons were performed to assess the disparities in the variables between the components. Substantial differences were assessed utilizing an analysis of variance (ANOVA) through a statistical software program (Statistix version 8.0). When a substantial difference was observed, the means were distinguished utilizing Tukey’s honestly significant difference (HSD) and least significant difference (LSD) tests at α = 0.05.

## 5. Conclusions

The results of this study led the researchers to conclude that the application of a soil spray containing four water extracts led to increased growth in the plants exposed to the spray. This study revealed a stimulatory response of the growth of fruity and leafy vegetables, as evidenced by increases in the plant heights of 41–68% for cucumber, 33–56% for tomato, 40–57% for kale and 42–60% for lettuce and the shoot fresh weights of 42–69% for cucumber, 40–64% for tomato, 46–65% for kale and 42–63% for lettuce from 1 WAS to 4 WAS of application times following the extract treatments. The increased photosynthesis, the total contents of phenols and flavonoids, and the higher activities of antioxidant enzymes probably contributed to the better growth of these plant species. The greenhouse experiments showed that the extracts of *P. guajava* and *A. sativum* were effective at promoting the growth of vegetables and increasing the production of secondary metabolites. To establish a link between the controlled greenhouse trials and real agricultural applications, it is important to evaluate the efficacy of these two extracts under different field conditions. This includes evaluating their performance in different soil types, climatic regions and crop species, while also considering the long-term environmental impact, residue safety and economic viability for sustainable agricultural practices. Field trials should investigate the integration of these extracts into standard cropping practices, including fertilization, irrigation and pest control, to confirm their efficacy and practicality for widespread use. Future field studies should evaluate the cost-effectiveness of these extracts to ensure economic viability, focusing on their ability to reduce the input costs, increase the yield and improve the crop quality. A comprehensive cost–benefit analysis comparing these natural extracts with current growth promoters will underpin their status as sustainable and economically beneficial alternatives.

## Data Availability

The data are contained within this article.
